# Biomarkers in Acute Myocardial Infarction Diagnosis and Prognosis

**DOI:** 10.5935/abc.20190131

**Published:** 2019-07

**Authors:** Paula F. Martinez, Silvio A. Oliveira-Junior, Bertha F. Polegato, Katashi Okoshi, Marina P. Okoshi

**Affiliations:** 1 Instituto Integrado de Saúde, Universidade Federal de Mato Grosso do Sul, Campo Grande, MS - Brazil; 2 Faculdade de Medicina de Botucatu - Departamento de Medicina Interna - Universidade Estadual Paulista (UNESP), Botucatu, SP - Brazil

**Keywords:** Cardiovascular Diseases, Diagnosis, Prognosis, Mortality, Biomarkers, Sirtuins, Myocardial Infarction, Molecular Biology

Biomarkers have become a helpful tool for clinicians to establish acute and chronic diseases diagnosis and prognosis more accurately. The rapid expansion of researches in this field has been stimulated by molecular biology and omics techniques development.^1^

In cardiovascular disease, the release of the intracellular components into the bloodstream in higher concentrations than usual are related to pathological conditions, such as necrosis, inflammation, hemodynamic stress, and thrombosis, and considered potential biomarkers.^2^ Although a large number of cardiac biomarkers has been described, only a few of them were incorporated in clinical practice. Their usefulness depends on their specificity and sensitivity for detecting myocardial injury, reproducibility, accuracy, and the discriminatory limits to distinguish between pathologic from physiologic levels.^2^

Cardiac troponin (cTn) is a biomarker that has been established for diagnosis and that also provides robust prognostic information in acute myocardial infarction (AMI).^1^ It is still the most recommended biomarker for detecting myocardial injury, especially due to its sensibility and specificity, even though it does not indicate the underlying etiology and pathophysiological mechanism.^3^ On the Fourth Universal Definition of Myocardial Infarction, myocardial infarction is defined when acute injury with abnormal cardiac biomarkers is detected (a rising and/or falling pattern of cTn values with at least one value above the 99^th^ percentile upper reference limit) in association with evidence of acute myocardial ischaemia.^3^

Several other biomarkers representing different pathophysiological axes have been considered as a potential tool for diagnosis and risk stratification in AMI patients. These emerging biomarkers, including suppression of tumorigenicity 2 (ST2), galectin-3, copeptin, myeloperoxidase (MPO), high sensitivity C-reactive protein (CRP), pregnancy-associated plasma protein A (PAPP-A), growth-differentiation factor-15 (GDF-15), and others, have been studied individually or using a multimarker strategy.^4,5^ However, further studies are still required to determine their usefulness for AMI diagnosis and prognosis.

More recently, sirtuins have attracted great interest for their protective roles against inflammation, cancer, cardiovascular disease, vascular aging, and glucose homeostasis changes. Sirtuins were first discovered in the 1970s; their main action is the removal of acetylated groups from histone and non-histone proteins in the presence of nicotinamide adenine dinucleotide (NAD^+^).^6^ Members of the sirtuin family are widely distributed throughout nature; this family has seven isoforms in the human body (Sirtuin1-7).^7^ Among sirtuins, the isoforms 1, 3, and 6 are the best characterized; they exert important effects in the cardiovascular system against atherosclerosis, myocyte hypertrophy, ischemia/reperfusion injury, oxidative stress, inflammation, and endoplasmic reticulum stress.^8-10^ Therefore, several studies have focused on the modulation of sirtuins with pharmacological and natural dietary compounds.^9^

In this issue of the Arquivos Brasileiros de Cardiologia, Kızıltunç et al.^11^ published an interesting study in which they hypothesized serum sirtuin 1, 3 and 6 levels as possible biomarkers of myocardial infarct size and prognosis in AMI patients. Temporal levels of serum sirtuin 1, 3 and 6 and their association with AMI prognostic markers were examined. Patients with AMI (n = 40) and patients with normal coronary arteries (n = 40) were included and evaluated regarding left ventricular ejection fraction (LVEF), serum proBNP, CRP, and serum levels of sirtuin1, sirtuin 3, and sirtuin 6. Peak troponin T, GRACE score, and first day/second-day sirtuin levels were recorded for AMI patients. The authors found that sirtuin 1, 3 and 6 levels in AMI were similar to those in normal coronary patients, and no temporal change in sirtuins levels was observed during infarction course. Furthermore, there was no significant correlation between sirtuins levels and traditional markers of infarct size such as proBNP, peak troponin T, or LVEF. However, baseline sirtuin 1 and 6 levels were positively correlated with reperfusion duration, and baseline sirtuin 3 was negatively correlated with GRACE score. As pointed out by the authors, the negative correlation between sirtuin 3 and GRACE score suggests a possible role of sirtuin 3 for risk assessment in AMI patients.

In this study, it should be pointed out that 95% of AMI patients were classified as Killip I functional class. Therefore, although the authors have observed similar sirtuins levels in AMI and normal coronary patients, further investigation is needed to clarify whether serum sirtuin levels modify in AMI patients with worse cardiac function.^12^

In the literature, experimental artery occlusion-mediated infarction was combined with decreased sirtuin 3 expression in rats.^13^ Furthermore, sirtuin 3 knockout induced coronary microvascular dysfunction and cardiac remodeling impairment, while sirtuin 3 upregulation improved cardiac function in post-infarction mice.^8^ On the other hand, sirtuin 3 deficiency did not change infarction size or cardiac function.^14^ Therefore, additional studies are needed to clarify the role of sirtuins in the pathophysiology of myocardial infarction.
